# Ultrasound-Guided Miniscalpel-Needle Release versus Dry Needling for Chronic Neck Pain: A Randomized Controlled Trial

**DOI:** 10.1155/2014/235817

**Published:** 2014-10-16

**Authors:** Yongjun Zheng, Dongping Shi, Xiaotong Wu, Minghong Gu, Zisheng Ai, Kun Tang, Le Ye, Xiangrui Wang

**Affiliations:** ^1^Department of Pain Management, Renji Hospital, School of Medicine, Shanghai Jiao Tong University, 1630 Dongfang Road, Shanghai 200127, China; ^2^Department of Anesthesiology, Shanghai Jiading Central Hospital, Shanghai 201800, China; ^3^Department of Anesthesiology, Shanghai First Rehabilitation Hospital, Shanghai 200090, China; ^4^Department of Pain Management, Shanghai Jiading Hospital of Traditional Chinese Medicine, Shanghai 201800, China; ^5^Department of Preventive Medicine, College of Medicine, Tongji University, Shanghai 200092, China; ^6^Department of Anesthesiology, Tongren Hospital, School of Medicine, Shanghai Jiao Tong University, Shanghai 200336, China

## Abstract

*Objective*. To compare ultrasound-guided miniscalpel-needle (UG-MSN) release versus ultrasound-guided dry needling (UG-DN) for chronic neck pain. *Methods*. A total of 169 patients with chronic neck pain were randomized to receive either UG-MSN release or UG-DN. Before treatment and at 3 and 6 months posttreatment, pain was measured using a 10-point visual analogue scale (VAS). Neck function was examined using the neck disability index. Health-related quality of life was examined using the physical component score (PCS) and mental component score (MCS) of the SF-36 health status scale. *Results*. Patients in the UG-MSN release had greater improvement on the VAS (by 2 points at 3 months and 0.9 points at 6 months) versus in the UG-DN arm; (both *P* < 0.0001). Patients receiving UG-MSN release also showed significantly lower scores on the adjusted neck disability index, as well as significantly lower PCS. No severe complications were observed. *Conclusion*. UG-MSN release was superior to UG-DN in reducing pain intensity and neck disability in patients with chronic neck pain and was not associated with severe complications. The procedural aspects in the two arms were identical; however, we did not verify the blinding success. As such, the results need to be interpreted with caution.

## 1. Introduction

Chronic neck pain is a common and disabling condition [1, 2]. It usually originates in discrete, hyperirritable focal spots (trigger points) in a taut band of skeletal muscle; onset of pain often occurs in conjunction with musculoskeletal disorders [3]. Injecting analgesic medication directly into trigger points could provide short-term relief of the symptoms [3]. However, several studies suggested that dry needling (DN; simply inserting a needle into trigger points) can also provide pain relief [4–6]. In fact, DN of primary trigger points has been shown to inhibit activity at satellite trigger points [7]. A meta-analysis, however, has concluded that DN does not provide significant therapeutic effect relative to placebo [8].

Miniscalpel-needle (MSN) release is a promising treatment for chronic neck pain [9–11]. In this technique, a small needle-scalpel is used to stimulate areas of chronic injury or soft tissue damage [9]. In one study [9], MSN release led to significantly greater reduction in pain intensity than did acupuncture, as well as significantly greater increases in pain threshold and motion range at 3 months after treatment [9].

A major concern of the conventional MSN release is the potential damage to nerves and blood vessels [12, 13]. Limited evidence suggests that performing the MSN procedure under the guidance of ultrasound could help to avoid damaging critical nerves and vessels in unexpected locations [14–16].

The current study is a randomized controlled trial (RCT) that compared MSN release and dry needling in patients with chronic neck pain.

## 2. Subjects and Methods

### 2.1. Subjects

This study was approved by the Institutional Review Board of Renji Hospital (Shanghai, China) and registered in the Chinese Clinical Trial Register (ChiCTR-TRC-10001609). Data collection, analysis, and reporting were performed in compliance with the CONSORT guidelines [17]. Written informed consent was obtained from all participants.

Consecutive adult (≥18 years old) outpatients with chronic neck pain seeking medical treatment at the Department of Pain Management, Renji Hospital, from January 2009 to February 2010 were recruited. Eligibility was determined at the preliminary interview. For inclusion in the study, patients had to meet all the following criteria: chronic neck pain for more than three months; a score > 3 on a 10-point visual analogue scale (VAS); and the presence of trigger points in the neck. Trigger points were established based on both physical signs (hypersensitive bundle or nodule of muscle fibers harder than normal upon palpation) and ultrasound imaging (hyperechoic skin, hyperechoic marbled appearance of the muscle, and mixed echogenicity). Patients were excluded if they were pregnant or if they had a history of acupuncture, DN, MSN release, or vertebral column surgery. They were also excluded if they had any of the following conditions: protrusion or prolapse of one or more intervertebral discs with concurrent neurological symptoms; infectious spondylopathy; chronic neck pain caused by inflammatory, malignant, or autoimmune disease; congenital deformation of spine except slight lordosis or scoliosis; compression fracture caused by osteoporosis; spinal stenosis and spondylolysis or spondylolisthesis.

### 2.2. Pain Management

Patients were randomly assigned to receive either UG-MSN release or UG-DN using a block design (*n* = 8). The allocation concealment was carried out using the SNOSE method (sequentially numbered, opaque, sealed envelopes). UG-MSN was performed using an MSN (Shenlong Medical Apparatus, Suzhou, China; see Supplementary Figures 1(a) and 1(b) in the Supplementary Material available online at http://dx.doi.org/10.1155/2014/235817) as described earlier [9, 10] (Supplementary Figure 2(a)). The ultrasound system (SonoSite, Bothell, Washington, USA) comprised a high-frequency linear array transducer (10–5 MHz) covered by a sterilized medical condom as a disposable sterile medical ultrasound sheath (Shanghai Medical Dressing Factory, Shanghai, China; Supplementary Figures 1(c) and 1(d)).

With the patient in a lateral decubitus position, trigger points were identified by palpation by one author (YJZ) and marked with indelible ink. The skin was sterilized by povidone iodine. The transducer was applied transversely to the lateral aspect of the neck to obtain a transverse axial view. The seventh cervical vertebra (C7) was used as a reference and identified by the rudimentary anterior tubercle and a prominent posterior tubercle on the transverse process (Figures 2(a)–2(c)) [16]. By moving the transducer cranially, C6 was easily identified (Figure 3). Trigger points were identified by the following features: hyperechoic skin, adipose tissue of mixed echogenicity, and hyperechoic marbled appearance of muscle (Figure 3(c)) [18]. After confirming the trigger points, intradermal anesthesia with 1% lidocaine was performed using a 25-gauge, 1.5-inch needle and 2 mL syringe, and either UG-MSN release or UG-DN was performed on each point.

One author (YJZ) performed the treatment (either MSN release or DN under ultrasound guidance). For MSN release, when the pinpoint needle reached the trigger point, the MSN was moved up and down 2-3 times longitudinally around the vertebral lamina and the posterior tubercle of the transverse processes of C6 (Figure 3(c)). The miniscalpel did not extend deeper than the posterior tubercle of the transverse processes of the cervical vertebrae, thereby avoiding important structures between the posterior and anterior tubercles of the cervical vertebrae [19]. MSN release was performed once for each trigger point. For UG-DN, the needle was inserted in the trigger point, longitudinal to the transducer at the level of C6 ((Supplementary Figure 2(b)) and Figure 4). Needle placement was verified by asking the patient to contract the muscle and observing the needle under ultrasound. The needle was moved up and down 2-3 times. Again, the needle was inserted no deeper than the posterior tubercle of the transverse processes of the cervical vertebrae. DN was performed once for each trigger point.

### 2.3. Outcomes

The primary outcome was pain intensity. Secondary outcomes included pain disability and health-related quality of life. Pain intensity was measured using the 10-point VAS [18]. Pain disability was measured using the neck disability index (NDI), consisting of 10 questions with six possible responses, each of which was assigned a different number of points. The total NDI score can range from 0 to 50 points, with 50 indicating the worst condition [20, 21]. Health-related quality of life was measured using the SF-36 health status scale [22, 23].

The survey instruments were administered by a pain management specialist blinded to the treatment (even whether a patient was in the study or not). Outcomes were assessed before the treatment and at 3 and 6 months after the treatment.

### 2.4. Sample Size

Sample size was calculated based on a preliminary RCT involving 60 patients with chronic neck pain, 30 of whom were treated by UG-MSN release and 30 by UG-DN. None of the 60 subjects in this preliminary study was included in the current study. Sample size (*n*) was calculated according to the following equation for repeated measures at a significance level of 0.05 and power of 80%: *n* = 2 × (*μ*_*α*_/2+*μ*_*β*_)^2^ × *δ* ^2^ × (1 − *ρ*)/(*m* × *s* ^2^ × (*β*_1*A*_−*β*_1*B*_)^2^)  (*μ*_*α*_/2 = 1.96, *μ*_*β*_ = 0.842) [24]. Based on the results of the preliminary study, the variance of repeated measurements (*δ* ^2^) was 3.08; the symmetric correlation coefficient (*ρ*) was 0.38; the number of repetitions (*m*) was 3; *s* ^2^ = ∑(*t*_*j*_−*t*)^2^/*m* = 8.25, where *t*_*j*_ is the time when repeated measures are performed and *t* is the average time of repeated measures; and *β*_1*A*_ − *β*_1*B*_ = 0.137, where *β*_1*A*_ and *β*_1*B*_ are the slopes (change in measurement over time) for the UG-MSN and UG-DN groups, respectively. This equation indicated the need for 64 participants per arm, so we recruited 80 patients for each arm to take into account a 20% dropout rate.

### 2.5. Randomization

Subjects were randomly allocated into UG-MSN or UG-DN group based on a computer-generated allocation sequence using a block design (*n* = 8). Allocation concealment was carried out using the SNOSE method (sequentially numbered, opaque, sealed envelopes). The next envelope in the sequence was opened after the participant had given informed consent.

### 2.6. Blinding

Patients were blinded to their treatment allocation and did not see the instruments during the procedure. The pain management specialist who conducted assessment was also blinded to the treatment condition. The same physician (YJZ) performed UG-MSN and UG-DN procedures on all participants. Data were analyzed by an independent statistician.

### 2.7. Statistical Analysis

All data were analyzed using SPSS 13.0 (IBM, Chicago, USA). Data for continuous variables showing a normal distribution were reported as means and standard deviations (SD); otherwise, data were reported as median. Intergroup differences were assessed for statistical significance using independent Student's *t*-test for normally distributed data and the Mann-Whitney test for nonnormally distributed data. Intergroup differences in categorical variables were assessed for significance using the *χ* ^2^ test or Fisher's exact test. Analysis of covariance was also used to assess differences between the two groups based on *F*-statistics. The baseline served as a covariate. The significance level for all tests was set at 0.05.

## 3. Results

### 3.1. Participant Flow

**Figure 1:**
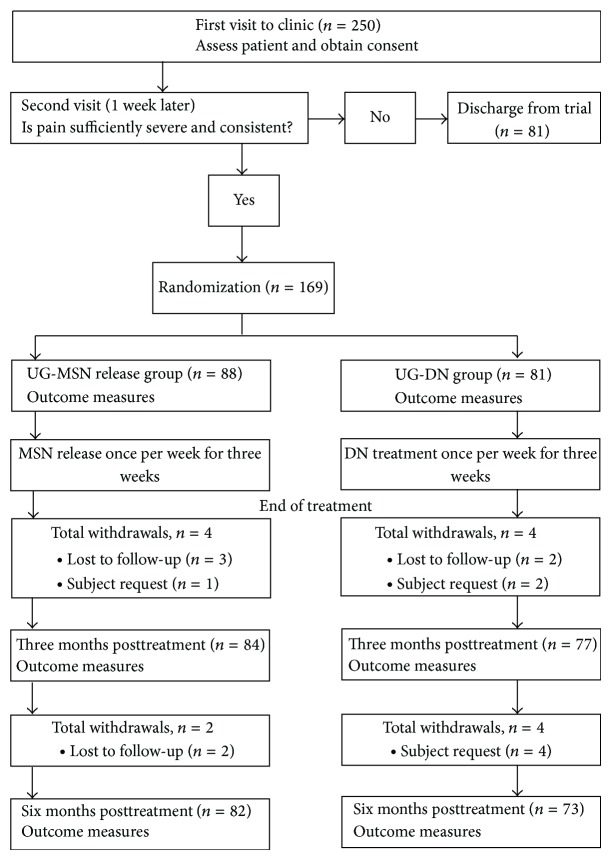
Schematic presentation of the study flow.

**Figure 2:**
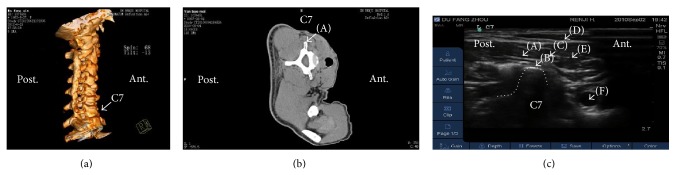
Ultrasound identification of the seventh cervical vertebra (C7) during the UG-MSN and UG-DN techniques. (a) Three-dimensional computed tomography reconstruction of the cervical vertebrae. The white arrow indicates C7. (b) Conventional computed tomography scanning of C7. (A) The posterior tubercle of the transverse process of C7. (c) Ultrasound imaging of C7. The dotted line represents the posterior tubercle of the transverse process of C7. The letters (A)–(F) indicate, respectively, the medial scalenus muscle, posterior tubercle, brachial plexus, sternocleidomastoideus muscle, anterior scalenus muscle, and cervical artery. Post.: posterior position of patients. Ant.: anterior position of patients.

**Figure 3:**
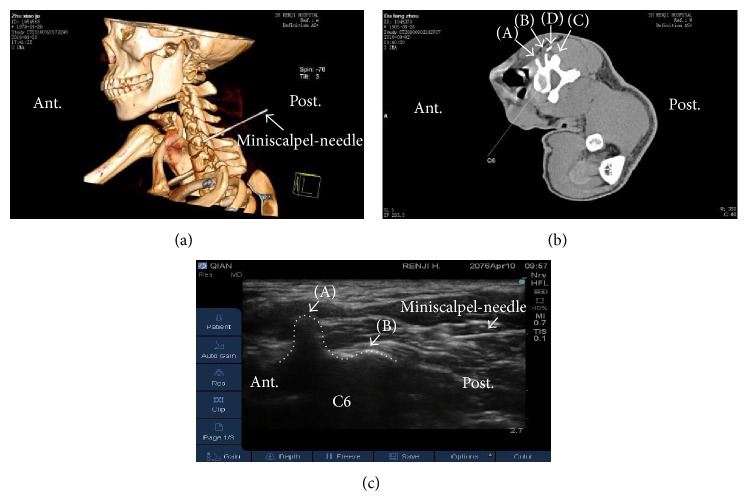
UG-MSN release at the sixth cervical vertebra (C6). (a) Three-dimensional computed tomography reconstruction of the puncture site at C6. The white arrow indicates the MSN. (b) Conventional computed tomography scanning of C6. The letters (A)–(D) indicate, respectively, the anterior tubercle, posterior tubercle, articular process, and the MSN. (c) UG-MSN release at C6, with the MSN clearly indicated. Trigger points showed the following characteristics: hyperechoic skin, adipose tissue of mixed echogenicity, and muscle of hyperechoic, marbled appearance. The dotted line shows the outline of C6. The letters (A) and (B) represent, respectively, the posterior tubercle and articular process. Post.: posterior position of patients. Ant.: anterior position of patients.

**Figure 4:**
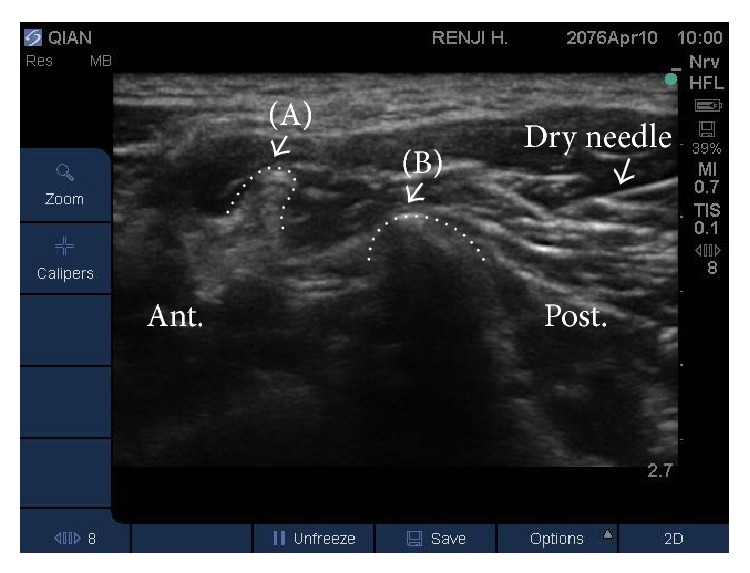
UG-DN at the sixth cervical vertebra (C6). The dry needle is clearly indicated. Trigger points showed the same characteristics described in Figure 3. The dotted line shows the outline of C6. The letters (A) and (B) indicate, respectively, the posterior tubercle and articular process. Post.: posterior position of patients. Ant.: anterior position of patients.

From January 2009 to February 2010, 250 participants were approached, of which 81 were excluded, leaving 169 who were randomized into UG-MSN or UG-DN group (Figure 1). Each group received the corresponding treatment once a week for three weeks. Of the 169 patients, 14 (8.3%) dropped out, and the dropout rate was similar for the UG-MSN release and UG-DN groups (6.8% versus 9.9%, *P* = 0.5801, *χ* ^2^ test). In the UG-MSN group, 4 patients were lost to follow-up by 3 months posttreatment and 6 were lost by 6 months. In the UG-DN group, 4 patients were lost by 3 months and 8 were lost by 6 months.

Scores on the VAS, NDI, SF-36 PCS, and SF-36 MCS were measured immediately prior to the start of treatment and again at 3 and 6 months posttreatment. VAS scores at baseline were similar for the patients lost to follow-up from either group at 3 months and 6 months (*P* > 0.05, Mann-Whitney nonparametric tests). This suggests that losses to follow-up did not significantly affect the outcomes measured for the patients who completed the trial.

### 3.2. Baseline Characteristics

Baseline demographic data, disease duration, previous treatments, and scores on the VAS, NDI, SF-36 PCS, and SF-36 MCS are shown in Table 1. None of these parameters differed significantly between the two treatment groups.

### 3.3. Outcome Measures

The final analysis included data for 82 of 88 patients (93.2%) in the UG-MSN release group and for 73 of 81 patients (90.1%) in the UG-DN group. Analyses of covariance were performed in which the score on the VAS, NDI, SF-36 PCS, or SF-36 MCS at 3 and 6 months served as the response variable (outcome) and treatment served as the explanatory variable; results were adjusted for baseline differences in the response variable.

At 3 months posttreatment, the adjusted mean VAS was 0.9 points higher in the UG-DN group than in the UG-MSN release group (*β* = 0.9, SD = 0.2, *P* < 0.0001); at 6 months, this difference had grown to 2.0 points (*β* = 2.0, SD = 0.2, *P* < 0.0001; Table 2). Group-level analysis showed that mean VAS decreased by 51.5% from baseline to 3 months in the UG-MSN release group, compared to 40.8% in the UG-DN group. Mean VAS decreased by 44.1% from baseline to 6 months in the UG-MSN release group, compared to 18.3% in the UG-DN group. Patient-level analysis showed that 50 of 84 patients in the UG-MSN release group showed a >50% reduction in VAS from baseline to 3 months, compared to 38 of 84 patients in the UG-DN group. The corresponding proportions at 6 months were 35 of 82 patients in the UG-MSN release group, compared to 15 of 82 patients in the UG-DN group.

At 3 months posttreatment, the adjusted average NDI was 2.4 points higher in the UG-DN group than in the UG-MSN release group (*β* = 2.4, SD = 0.7, *P* < 0.0001); at 6 months, this difference had grown to 5.3 points (*β* = 5.3, SD = 0.7, *P* < 0.0001; Table 2). Group-level analysis showed that mean NDI decreased by 64.3% from baseline to 3 months in the UG-MSN release group, compared to 45% in the UG-DN group. Mean NDI decreased by 60.3% from baseline to 6 months in the UG-MSN release group, compared to 23.8% in the UG-DN group. Patient-level analysis showed that 55 of 84 patients in the UG-MSN release group showed a >50% reduction from baseline to 3 months, compared to 40 of 84 patients in the UG-DN group. The corresponding proportions at 6 months were 50 of 82 patients in the UG-MSN release group, compared to 17 of 82 patients in the UG-DN group.

At 3 months posttreatment, the adjusted mean SF-36 PCS was 5.5 points lower in the UG-DN group than in the UG-MSN release group (*β* = 5.5, SD = 2.2, *P* = 0.013); at 6 months, this difference had grown to 7.5 points (*β* = 7.5, SD = 3.3, *P* = 0.024; Table 2). Group-level analysis showed that mean SF-36 PCS improved by 19.1% from baseline to 3 months in the UG-MSN release group, compared to 1.1% in the UG-DN group. Mean SF-36 PCS improved by 33.4% from baseline to 6 months in the UG-MSN release group, compared to 9.9% in the UG-DN group. However, the two treatment groups showed similar SF-36 MCS scores at 6 months.

### 3.4. Treatment Adherence

Treatment adherence rates were similar between the two treatment groups: 82 of 88 subjects (93.2%) in the UG-MSN release group completed the study protocol, compared to 73 of 81 (90.1%) in the UG-DN group (*P* = 0.5801, *χ* ^2^ test).

### 3.5. Adverse Events

Six participants in the UG-MSN release group (7.3%) and 7 in the UG-DN group (9.6%) reported mild reactions during treatment, mainly slight pain and somatic reactions such as sweating. However, all these participants agreed to continue the treatment. No serious adverse complications, such as spinal injury or nerve injury, were recorded.

## 4. Discussion

The results of this randomized trial suggest that UG-MSN release is more effective than UG-DN in reducing pain intensity and neck disability and in increasing health-related quality of life in patients with chronic neck pain. Both treatments appear to be associated with similarly low rates of mild adverse events, similar dropout rates, and no severe side effects. These findings should be confirmed in larger cohorts. To our knowledge, this is the first report using ultrasound to guide MSN release.

The greater efficacy of UG-MSN may reflect the fact that it combines the therapeutic action of DN and minimally invasive surgery. MSN release can cut and detach the taut band in myofascial pain syndrome as well as relaxing the compressed blood vessels and trapped nerves at the primary and second foci [25]. MSN release also stimulates vessel and nerve bundles, restores mechanical dynamic equilibrium, and improves local microcirculation [25]. In addition to avoiding damage to important adjacent structures, ultrasound imaging could enhance the accuracy of the needle placement and probably contributed to the apparently superior efficacy of the treatment.

DN and MSN have been shown to relieve chronic neck pain [3, 26], but proper positioning of trigger points is essential to the success of both techniques and for avoiding severe injuries, including pneumothorax and intercostal nerve damage [12, 13, 27]. Trigger points are sometimes difficult to palpate during physical examination, especially in obese patients, and using ultrasound to guide trigger point injections has been linked to greater therapeutic efficacy and lower possibility of complications [28]. Our results extend these findings by showing for the first time that ultrasound guidance can be used safely and effectively to perform MSN.

Our finding that UG-MSN release is superior to UG-DN is reminiscent of a previous study showing MSN release to be superior to acupuncture in managing myofascial pain syndrome [9]. Our results compared an experimental treatment (UG-MSN) with an active control (UG-DN), and we expect that the therapeutic advantage of UG-MSN would be even greater if a placebo control group had been used. An inactive control group, however, may be unethical under these circumstances, and the debate about ideal controls for acupuncture studies continues [29]. We ruled out using a control group that would receive MSN release without ultrasound guidance because of the risk of severe complications; we also ruled out a control group that would receive UG-MSN release outside the trigger points because the resulting lack of efficacy might drastically reduce compliance. Risk of complications led us to rule out control groups treated by other approaches, including acupuncture, steroid injection, and trigger point injection [9–11].

In addition to its randomized and controlled nature, our study fulfills the essential requirement of using well-validated outcome measures. While outcomes based on self-report inherently carry some risk of bias, we believe that outcomes like pain, functional ability, and quality of life can be more accurately assessed by listening to patients rather than physicians.

All treatments were conducted by a single physician in the current study. Such a design helped to ensure consistency between the two treatment groups but decreased the generalizability of the results. Another concern is the completeness of the blinding: patient experience during the procedure may help them to identify which treatment they received. We included only “naïve” subjects in the current study: those who received acupuncture or miniscalpel-needle release previously were not included. Nonetheless, we did not take solid measures to verify the blinding success. As such, the results of the current study need to be interpreted with caution.

## Supplementary Material

Supplementary Figure 1. A. A schematic diagram of a miniscalpel-needle (MSN). B. A photohragph of a MSN. C. The high-frequency liner array transducer (upper) and disposal sterilizing medical ultrasonic coupling agent (subjacent). D. Ultrasound device.
Supplementary Figure 2. A. The procedure of UG-MSN release. B. The procedure of UG-DN.

## Figures and Tables

**Table 1:** Baseline characteristics of patients with chronic neck pain before random allocation to be treated with ultrasound-guided MSN release or dry needling.

Variable	UG-MSN release (*n* = 82)	UG-DN (*n* = 73)	*P* ^*^
Demographics			
Age, yr	39.0 ± 14.1	42.4 ± 13.7	0.1
Male, %	54.9	65.8	0.3
Disease duration, yr	4.7 ± 2.3	5.3 ± 2.9	0.1
Previous treatments, %			
Current analgesic medication	42.7	54.8	0.2
Physical therapy, chiropractic, or massage	47.6	58.9	0.2
Pain intensity			
10-point visual analog scale score	6.8 ± 1.6	7.1 ± 1.8	0.3
Neck disability			
Neck disability index score	17.9 ± 8.3	16.0 ± 5.4	0.1
Health-related quality of life (SF-36)			
Physical component score	41.3 ± 14.0	43.6 ± 16.1	0.1
Mental component score	43.4 ± 12.6	44.4 ± 16.8	0.7

UG-DN: ultrasound-guided dry needling; UG-MSN: ultrasound-guided miniscalpel-needle.

Data expressed as mean ± SD or %.

^*^Assessed using independent Student's *t*-test for continuous variables and using the *χ* ^2^ test or Fisher's exact test for categorical variables.

**Table 2:** Longitudinal comparison of primary outcomes in patients with chronic neck pain treated with either UG-MSN release or UG-DN.

Outcome	Baseline	3 months	6 months
Visual analog scale (VAS)			
UG-MSN release	6.8 ± 1.6	3.3 ± 1.2	3.8 ± 1.2
UG-DN	7.1 ± 1.8	4.2 ± 1.5	5.8 ± 1.4
^*^ *P* value		<0.0001	<0.0001
Neck disability index (NDI)			
UG-MSN release	17.9 ± 8.3	6.4 ± 3.7	7.1 ± 2.6
UG-DN	16.0 ± 5.4	8.8 ± 4.5	12.2 ± 5.5
^*^ *P* value		<0.0001	<0.0001
SF-36 physical component score (PCS)			
UG-MSN release	41.3 ± 14.0	49.2 ± 14.1	55.1 ± 19.0
UG-DN	43.6 ± 16.1	44.1 ± 13.6	47.9 ± 21.5
^*^ *P* value		0.013	0.024
SF-36 mental component score (MCS)			
UG-MSN release	43.4 ± 12.6	49.4 ± 14.6	51.4 ± 14.5
UG-DN	44.4 ± 16.8	49.0 ± 15.2	50.8 ± 16.7
^*^ *P* value		0.778	0.801

Data are expressed as mean ± SD.

^*^Based on analyses of covariance in which the primary outcome was the response variable and treatment was the explanatory variable; results were adjusted for baseline differences in the response variable.
