# Supplementary material for: Ultrasound-Guided Miniscalpel-Needle Release versus Dry Needling for Chronic Neck Pain: A Randomized Controlled Trial
Source: Evid Based Complement Alternat Med. 2014 Oct 16;2014:235817. doi: 10.1155/2014/235817 (PMC4214050; doi:10.1155/2014/235817)
Supplement: Supplementary file 1 — Supplementary Figure 1. A. A schematic diagram of a miniscalpel-needle (MSN). B. A photohragph of a MSN. C. The high-frequency liner array transducer (upper) and disposal sterilizing medical ultrasonic coupling agent (subjacent). D. Ultrasound device. Supplementary Figure 2. A. The procedure of UG-MSN release. B. The procedure of UG-DN. [file 235817.f1.pdf]

Supplementary figures

Supplementary Figure 1. The equipment used to treat chronic neck pain.

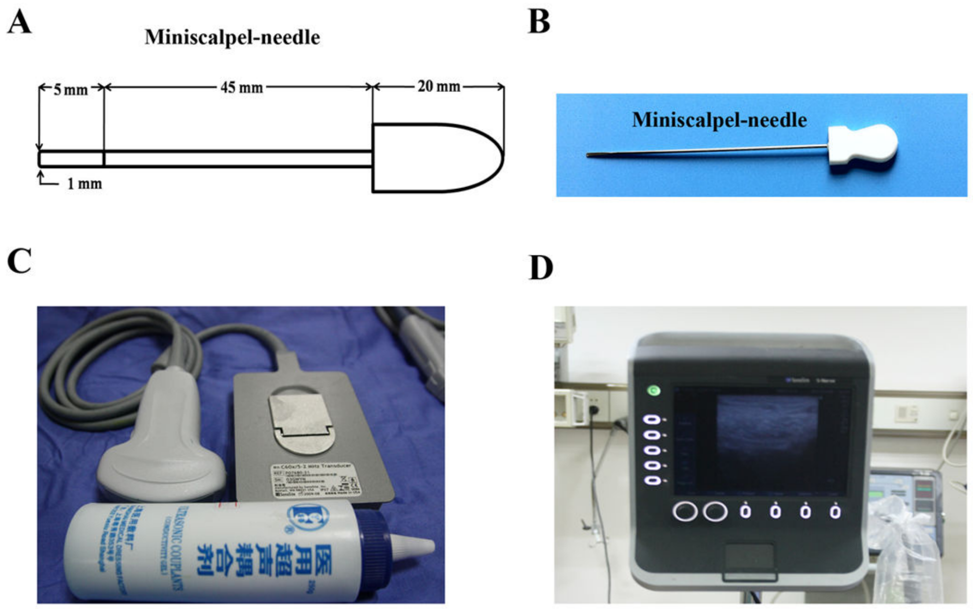

A. A schematic diagram of a miniscalpel-needle (MSN). B. A photohragph of a MSN. C. The high-frequency liner array transducer (upper) and disposal sterilizing medical ultrasonic coupling agent (subjacent). D. Ultrasound device.

Supplementary Figure 2. The procedure of UG-MSN release (A) and UG-DN (B).

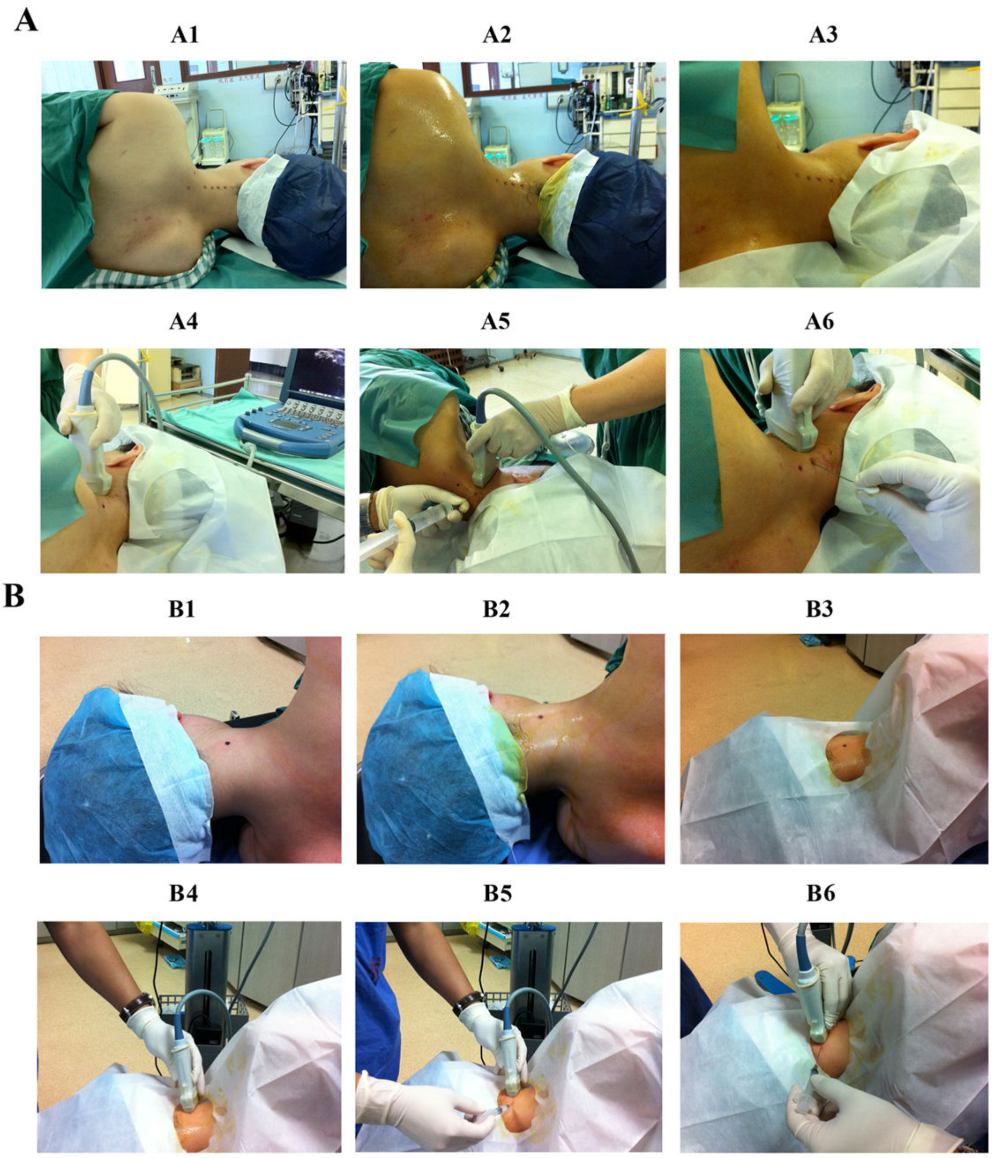

A. The procedure of UG-MSN release. A1. Trigger points were identified by palpation and marked with indelible ink. A2. The skin was sterilized by povidone iodine. A3. A sterile surgical towel was placed on the patient. A4. The trigger points were determined by ultrasound. A5. Intradermal anesthesia was performed on the trigger points. A6. UG-MSN release was performed on the trigger points. B. The procedure of UG-DN. B1. Trigger points were identified by palpation and marked

with indelible ink. B2. The skin was sterilized by povidone iodine. B3. A sterile surgical towel was placed on the patient. B4. The trigger points were determined by ultrasound. B5. Intradermal anesthesia was performed on the trigger points. B6. UG-DN was performed on the trigger points.
